# Stereotactic Microwave Ablation of Hepatocellular Carcinoma: The Impact of Tumor Size and Minimal Ablative Margin on Therapeutic Success

**DOI:** 10.3390/tomography9010005

**Published:** 2022-12-26

**Authors:** Antonia-Maria Pausch, Tamam Ghali, Tobias Wertheimer, Florian Zeman, Karolina Mueller, Michael Christian Doppler, Ingo Einspieler, Lukas Philipp Beyer, Stephan Schleder, Christian Stroszczynski, Lukas Luerken

**Affiliations:** 1 Department of Radiology, University Hospital Regensburg, 93053 Regensburg, Germany; 2 Institute of Diagnostic and Interventional Radiology, University Hospital Zurich, 8091 Zurich, Switzerland; 3 Department of Internal Medicine III, Hematology and Oncology, University Hospital Regensburg, 93053 Regenburg, Germany; 4 Center for Clinical Studies, University Hospital Regensburg, 93053 Regensburg, Germany; 5 Department of Diagnostic and Interventional Radiology, Medical Center-University of Freiburg, 79106 Freiburg, Germany; 6 Department of Diagnostic and Interventional Radiology, Klinikum Ernst von Bergmann, 14467 Potsdam, Germany; 7 Department of Diagnostic and Interventional Radiology, Merciful Brothers Hospital St. Elisabeth, 94315 Straubing, Germany

**Keywords:** interventional oncology, stereotactic navigation, microwave ablation, hepatocellular carcinoma

## Abstract

Background: Microwave ablation (MWA) has gained relevance in the treatment of hepatic malignancies and especially in hepatocellular carcinoma (HCC), and it is an important alternative to surgery. The purpose of the study was to evaluate whether the minimal ablative margin (MAM) or the initial tumor size has a greater effect on the success of stereotactic MWA of HCC regarding the time to local tumor progression (LTP) and overall survival (OS). Methods: 88 patients, who received stereotactic MWA of 127 tumor lesions with a curative intention were included in this single-center, retrospective study. The MAM was evaluated in a side-by-side comparison of pre- and post-ablative, contrast-enhanced slice imaging. A Cox proportional hazard model with a frailty term was computed to assess the influence of the MAM and the maximum tumor diameter on the time to LTP and the OS. Results: The maximum tumor diameter was identified as a significant positive predictor for LTP (hazard ratio 1.04, 95% CI 1.00–1.08, *p* = 0.03), but it was not a significant positive predictor for the OS (*p* = 0.20). The MAM did not have a significant influence on LTP-free survival (*p* = 0.23) and OS (*p* = 0.67). Conclusion: For the successful stereotactic MWA of HCC, the MAM and maximum tumor diameter might not have an influence on the OS, but the maximum tumor diameter seems to be an independent predictor of the time to LTP.

## 1. Introduction

Thermal ablation methods are increasingly recognized being as an integral part of guideline-oriented therapy for primary hepatic tumors and liver metastases. Especially for hepatocellular carcinoma (HCC), thermal ablation treatment has become a minimally invasive method, which is already on par with surgical procedures in early tumor stages. The indications for thermal ablation are determined in the European Association for the Study of the Liver (EASL) guidelines for the treatment of HCC (2018 version) [1].

The recently revised German S3 guideline for the diagnosis and treatment of hepatocellular and biliary carcinoma reinforced the role of ablative therapy in HCC. In tumors ≤ 3 cm, ablation and resection are considered to be equally effective methods. Moreover, especially in HCC < 3 cm in locations in which it is difficult to conduct resection or patients with impaired liver function, thermal ablation is now recommended as a first-line therapy [2].

A considerable difference between resection and ablation is that the progression of liver dysfunction influences the prognosis after the resection. In contrast to that, the prognosis after ablation seems to be dependent on the rapid decline in primary efficacy with an increasing tumor size; this is especially relevant for tumors that are larger than 3 cm [3].

Nevertheless, even the ablation of larger tumors can be performed due to technical improvements such as the use of multi-applicator systems and modern navigation systems [4,5,6,7,8,9,10,11]. Several studies could demonstrate efficacy rates of more than 80% for ablation of HCC ≥ 5 cm, with the 1 year survival rates being above 80% [12,13,14].

Radiofrequency ablation (RFA) and microwave ablation (MWA) are the two recommended techniques of thermal ablative therapy in HCC [1,2]. Whilst RFA is the most widely performed modality so far, MWA is increasingly becoming the preferred option [15]. This is also the case for the Department of Radiology at the University Hospital Regensburg, therefore, we focused on the microwave ablation of HCC lesions for our study.

Modern navigation systems can help to determine a suitable and safe trajectory. With the surrounding structures being at risk, the tissue properties and a minimum ablative margin (MAM) in the case of curative therapy have to be taken into consideration [16]. Post-procedural imaging usually serves to evaluate the treatment success and to rule out any kind of ablation-associated complication. Nevertheless, the imaging mostly shows the region of the induced treatment effect, while microscopic tumor residuals cannot be detected with standard imaging. Hence, the required MAM must be verified in all three dimensions to ensure that complete tumor destruction has occurred and to avoid local tumor progression (LTP). The literature recommends an MAM of 5–10 mm for RFA of liver lesions [17,18,19,20,21,22,23,24,25], but the “ideal” margin size for MWA of liver lesions has not yet been determined, especially when navigation systems are used for the planning of antenna trajectories and the placement of ablation antennas. Only one study analyzed a collection of patients with single nodule HCC ≤ 3 cm, who were treated with ultrasound-guided or conventionally CT-guided RFA or MWA [26]. The study did not find a difference between RFA and MWA for the recommended MAM of 5 mm.

Another discussed predictor for LTP is the tumor size, but while there are sufficient data on the negative correlation between the tumor size and overall survival (OS) for RFA of HCC [27,28,29,30,31], it remains unclear whether the tumor size is a determinant of OS and time to LTP after the MWA of HCC [31,32,33].

The purpose of our study was to evaluate whether the MAM or the initial tumor size has a greater effect on the success of stereotactic MWA of HCC regarding time to LTP and OS.

## 2. Materials and Methods

### 2.1. Patient Selection

This retrospective, single-center study was approved by the local ethics committee.

**Figure 1:**
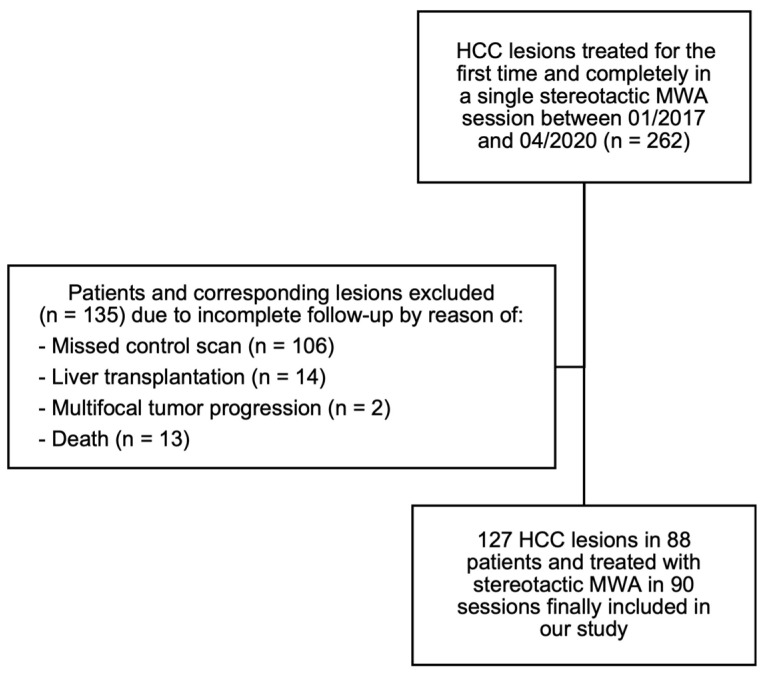
Flowchart of HCC lesion identification using in-/exclusion criteria for the final analysis of minimal ablative margin and maximum tumor diameter.

The indication for MWA was confirmed by an interdisciplinary tumor board for all the procedures. Before the MWA, all the patients were provided with comprehensive details and risks of the procedure, and informed consent was obtained. The ablation procedure was defined as successful if the ablation zone completely covered the margins of the original tumor lesion at the end of the ablation procedure. Technical failure, and thus, a residual tumor were defined as an HCC-typical arterial enhancement or wash out in the portal venous phase close to the borders of the ablation area in the control scan at the end of the ablation procedure or in control imaging at the first day following the ablation. Primary technical efficacy (PTE) was achieved if the first follow-up imaging showed no residual vital tumor tissue [16]. The data were collected retrospectively and consecutively from a database of patients with HCC who were treated by stereotactic MWA in a curative intention at our institution between 01/2017 and 04/2020. The included HCC lesions should be ablated for the first time and completely in a single session, thus, the patients who underwent re-ablations of previously ablated lesions and ablations with technical failure or ablations, where a tumor lesion was treated in more than one ablation session, i.e., the tumor was too large to be treated in one session, were excluded from the study. We evaluated the treated tumors regarding the LTP with follow-up imaging for at least 12 months (Figure 1). If the patients received a liver transplant (*n* = 14), died because of other reasons than tumor progression, complications from the intervention or complications from liver cirrhosis (*n* = 13), had a diffuse multifocal tumor progression, which made the follow-up regarding the LTP impossible (*n* = 2) or missed the follow-up imaging-appointments (*n*= 106) within the minimally required follow-up period, the corresponding lesions were excluded from the study. A flowchart of the patient selection process is provided in Figure 1, we included 127 HCC lesions in 88 patients who were treated in 90 stereotactic MWA sessions.

### 2.2. Stereotactic Microwave Ablation Procedure and Imaging

All the MWA treatments were performed under stereotactic guidance and general anesthesia. Using sterile radiopaque reflective optical markers attached to the patient after sterile preparation, a dual-phase contrast-enhanced planning CT (SOMATOM Definition Edge, Siemens Healthineers AG, Erlangen, Germany) was performed with an arterial and portal venous phase after the intravenous pressure injection of 120 mL of a non-ionic iodized contrast agent (Accupaque 350, GE Healthcare, Chicago, IL, USA). To avoid changes occurring in the liver position due to respiratory movement, the CT scans were acquired with the patient in temporary apnea. The CT data were transferred to the navigation system (CASOne IR, CAScination AG, Bern, Switzerland) adjacent to the CT gantry to define the ablation trajectories. The probes were then introduced through the CAScination aiming device. Before the ablation, an unenhanced CT scan was performed for the verification of the correct antenna position, and if this was necessary, the antenna position was manually corrected. Another control scan in the arterial and portal venous phase was obtained after the ablation was completed and the antenna were removed to rule out any peri-interventional complications and validate that a complete ablation had been performed. If a second contrast agent injection was not possible due to impaired renal function, a native control scan was performed to rule out peri-interventional complications, and CEUS (contrast-enhanced ultrasound) and native MRI on the first postinterventional day were performed to validate the ablation success.

Follow-up imaging was performed 6 weeks, 3 months, and after that, every 3 months following the MWA for 2 years. Two years after the MWA, the follow-up intervals were increased to 6 months. If it was possible, an MRI scan was performed using gadoxetic acid as a contrast agent (Primovist, Bayer AG, Leverkusen, Germany) and CareBolus with arterial (after 10 s), late arterial (after 40 s), portal venous (after 75 s) and hepatobiliary late phases (after 20 min). In rare cases, a CT scan was performed due to contraindications for MRI (having an implanted pacemaker or an allergy to MRI contrast medium, etc.). LTP was defined as a new lesion with an HCC-typical contrast enhancement pattern in association with the ablation area within a distance of less than 5 mm.

### 2.3. Evaluation of the Ablation Margin

**Figure 2:**
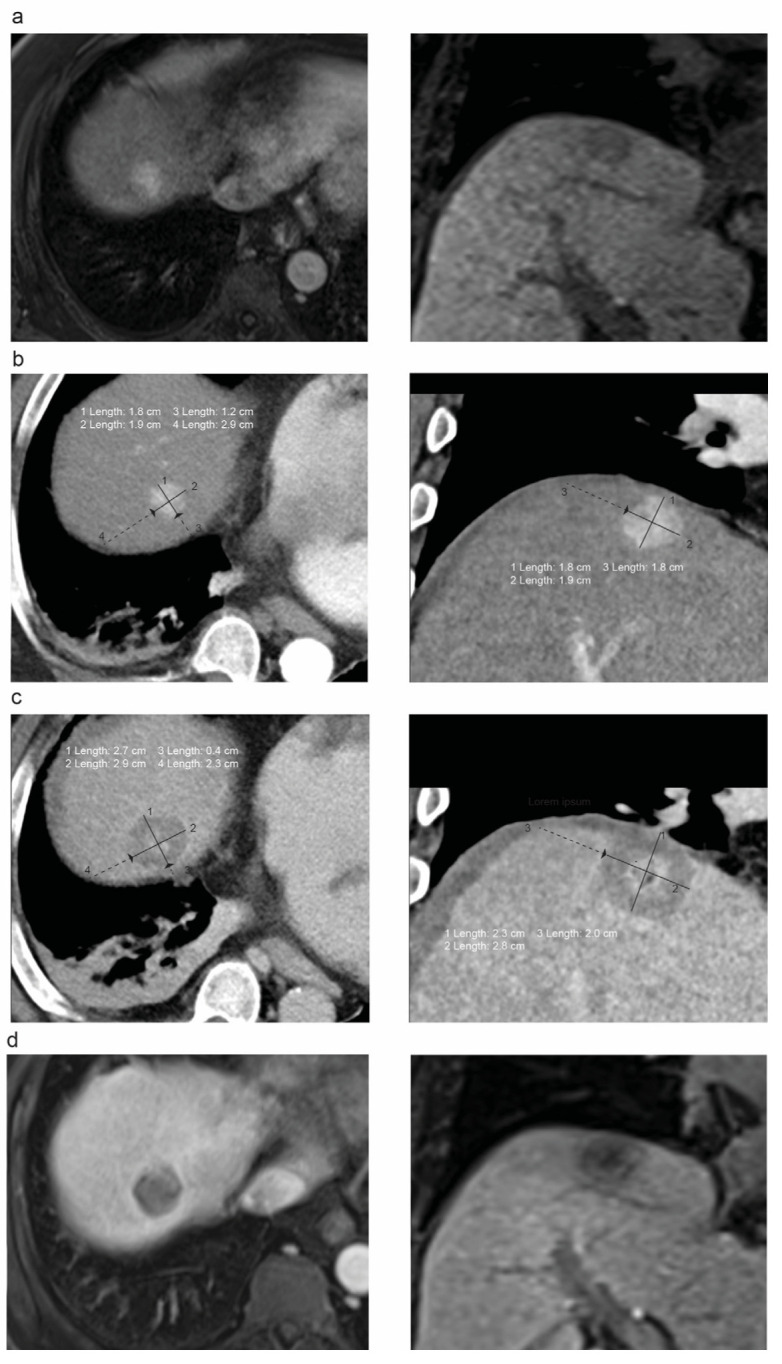
Example of minimal ablative margin assessment in MRI/CT scans of a 67-year-old man with HCC: (**a**) hepatic contrast-enhanced MRI (left: arterial phase, right: hepatobiliary phase) before MWA treatment; (**b**) contrast-enhanced planning CT scan (arterial phase) with pre-interventional measurements as performed in our study; (**c**) contrast-enhanced control CT scan (portal venous phase) after complete tumor ablation with postinterventional measurements; (**d**) contrast-enhanced follow-up MRI (left: arterial phase; right: hepatobiliary phase) 6 months after MWA with no evidence for local tumor progression.

The imaging evaluation was performed at a PACS workstation with split monitor capacities using syngo imaging (Siemens Healthineers AG, Erlangen, Germany). The two participating radiologists were blinded to the patients’ oncological outcome, and they evaluated the scans individually and independently. The CT or MRI datasets, in which the tumor lesion before the MWA and the ablation zone after the MWA showed the best visibility, were chosen. CEUS images were not used for the measurements due to a lack of comparability to CT and MRI images and a lack of standardization in the acquisition of CEUS images. Anatomic landmarks at the same axial scan level, which were visible on both the pre- and post-procedural scans, were identified. These were, for example, intrahepatic vessel bifurcations, focal intrahepatic lesions such as calcifications or surgical staples and undulations of the liver contour. Landmarks close to the lesion/ablation zone were preferred to correlate the pre- and post-ablation scans. The maximum and minimal ablation margin distance values were identified by subtracting the single values of the distances between the ablation zone/tumor border and the landmarks, respectively, in the six possible directions (medial, lateral, ventral, dorsal, cranial and caudal). Figure 2 visualizes the method for minimal ablation margin assessment.

### 2.4. Statistical Analysis

The interrater reliability was tested with the intraclass correlation coefficient (ICC). Due to nested data, i.e., some patients having several ablated tumor lesions, we computed a Cox proportional hazard model with a frailty term using SAS (SAS Version 9.4, SAS Institute Inc., Cary, NC, USA). SPSS Version 22 (IBM, New York, NY, USA) was used for all the other statistical analyses. A *p*-value < 0.05 was considered to be statistically significant.

## 3. Results

We analyzed 88 patients with a total of 127 HCC lesions that were ablated with stereotactic MWA in 90 sessions. The patient characteristics are summarized in Table 1. Between one and three HCC lesions were ablated per patient and session, however, in 61% of the cases, only a single HCC lesion was treated per patient and session.

The ICC for a two-way mixed effects model using an absolute agreement definition was 0.92 (95% CI 0.90–0.94). Thus, very good interrater variability of the two independent readers in measuring the investigated diameters and safety margins can be assumed [34]. On average, the maximum tumor diameter was 19.9 +/− 10.3 mm, which ranged from 0.6 cm to 5.3 cm, and the mean minimal ablative margin was 4.2 +/− 4.2 mm, which ranged from 0.0 cm to 2.4 cm.

**Figure 3:**
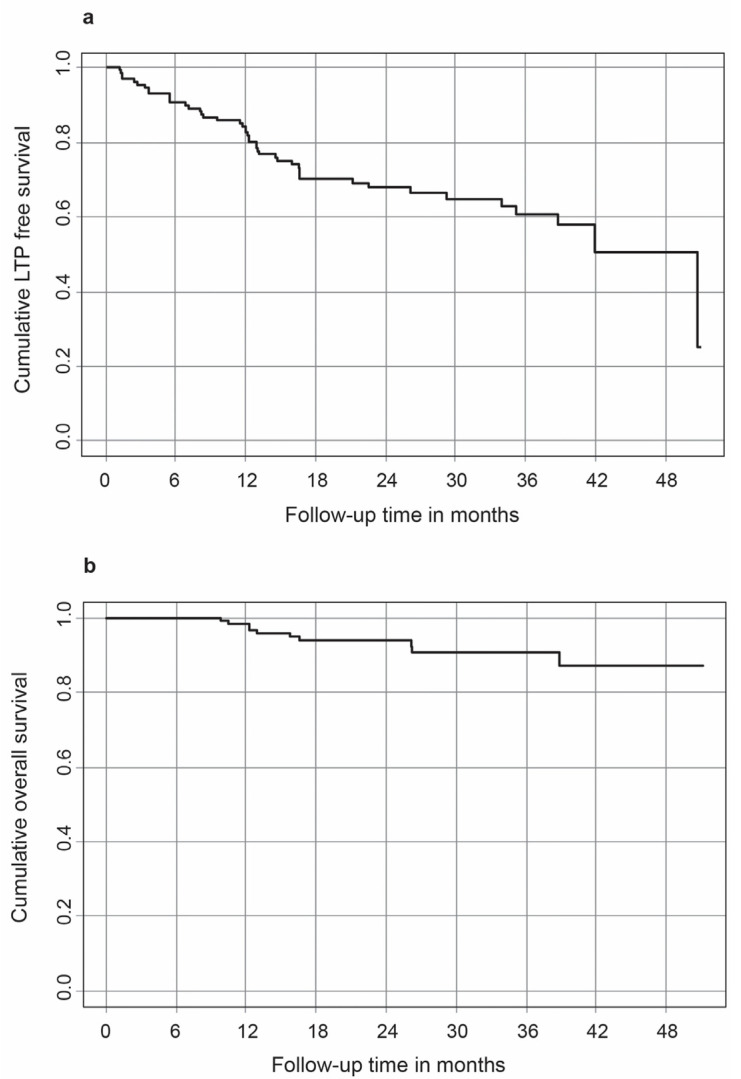
Kaplan–Meier curves for visualizing the probability of LTP free survival (**a**) and OS over time (**b**).

PTE was achieved in all the patients. The mean follow-up period was 25.8 +/− 11.6 months. In 37 out of 90 sessions (41.1 %), respectively, LTP was observed during the follow-up in 39 out of 127 HCC lesions (31.0 %). The LTP-free survival rates and OS rates for 1, 2 and 3 years post-ablation were 82.6%, 67.9% and 60.7% and 98.4%, 94.0% and 90.8%, respectively. Kaplan–Meier curves visualize the probability of LTP-free survival and OS over time (Figure 3).

A total of 12 complications occurred in 10 patients during the follow-up. Only one complication (1.1%), a rather extensive hematoma in the upper abdomen, was considered to be major (grade 3 according to the CIRSE classification system for complications). No immediate intervention was required, but the level of care and surveillance consequently increased. All the other 11 complications or ablation-associated side effects (12.2%) were classified as being minor (grade 1 and 2). No procedure-related deaths were observed. Table 2 presents an overview over the main features of the ablated HCC and the results of the follow-up.

Further, in our study we investigated an independent predictor for LTP-free survival and OS. The Cox proportional hazard model with a frailty term identified the maximum tumor diameter as a significantly positive predictor for LTP (hazard ratio 1.04, 95% CI 1.00–1.08, *p* = 0.032), but this was not the case for OS (*p* = 0.200). The minimal ablative margin did not have a significant influence on LTP-free survival (*p* = 0.23) or OS (*p* = 0.67).

## 4. Discussion

The aim of our study was to investigate the influence of the tumor size and ablation margin on the outcome of stereotactic MWA for the treatment of HCC regarding time to LTP and OS. While the initial PTE was achieved for all the lesions, 39 out of 127 ablated lesions showed LTP (31.0 %) during the follow-up with 1, 2 and 3 year LTP-free survival rates of 82.6%, 67.9% and 60.7%. Our analysis suggests that for the technically successful stereotactic MWA of HCC, neither the initial tumor size nor the MAM have a significant influence on OS, while the initial maximum tumor diameter seems be a better predictor for time to LTP than the MAM does.

Our study is, to our knowledge, the first study to evaluate and compare the impact of the tumor size and the MAM on the outcome of stereotactic MWA for HCC, but a few previous studies have analyzed similar issues in slightly different settings. The results of those studies are partially in contrast to our results. For example, Laimer et al. described that the MAM seems to be an independent predictor for LTP in stereotactic RFA for HCC, while the tumor size had no significant influence on LTP [35]. Wang et al. described that both the MAM and the tumor size are significant predictors for LTP after RFA for colon cancer liver metastases [19]. Another study by Li et al. demonstrated a significant correlation of tumor size and MAM with LTP for the thermal ablation of single nodule HCC ≤ 3 cm [26]; in this setting, there was no statistic difference between RFA and MWA regarding the ablation outcome and the recommendable MAM. Why those studies came to different conclusions than our study did remains unclear for the moment. Possible explanations for the different results of the studies could be the different characteristics of heat generation and distribution in MWA and RFA and, at least in comparison to Wang, the different tumor characteristics of HCC and colorectal liver metastases. Another possible explanation for the differing results could be the use of a stereotactic navigation system in combination with MWA because those methods seem to achieve better results regarding PTE and, respectively, OS in larger HCC lesions [32,36,37] settings, where conventional percutaneous ablation techniques show an incline in PTE and prognosis [3]. Further studies regarding these questions would be of interest.

The retrospective nature of our study as well as the exclusion criteria are limitations due to the possibility of selection bias. To minimalize this sort of bias, we tried to limit the exclusion criteria to a reasonable minimum by only excluding patients, for whom no sufficient evaluation regarding LTP could be performed, i.e., missing the follow-up imaging-appointments or receiving a liver transplant within 12 months of the ablation. Another limitation of our study is the fact that the ablation margin assessment was carried out visually and without the help of fusion software. A previous study from our department suggests that side-by-side comparison of the scans for the evaluation of safety margins might have poor reliability [38]. Nevertheless, we have chosen this type of analysis because this approach still comes closest to the reality of everyday clinical practice. Furthermore, in contrast to the results of the abovementioned study, ICC showed very good interrater reliability for our study. However, advances in the development of segmentation software may help to identify the peri- and post-ablation monitoring of ablation success in the future [39]. Accordingly, a software solution that is user friendly and fast enough to allow a reliable analysis of an ablation success to be made during the ongoing intervention would be of great interest. This is especially true for CT-guided ablations.

## 5. Conclusions

For successful stereotactic MWA of HCC, the MAM and maximum tumor diameter might not have an influence on the OS, but the maximum tumor diameter seems to be an independent predictor of the time to LTP.

## Figures and Tables

**Table 1:** Characteristics of 88 patients with a total of 127 HCC lesions treated with stereotactic MWA.

Characteristics	Value
*Patients (n in total)*	88
Female, *n* (%)	6 (6.7)
Male, *n* (%)	84 (93.3)
Age in years (range)	65 (9.3 (43–95)
*Child-Pugh score, n (%)*	
Child A	67 (76.1)
Child B	21 (23.9)
*BCLC-Classification, n (%)*	
BCLC 0 and A	55 (62.5)
BCLC B	29 (33.0)
BCLC C	4 (4.5)

**Table 2:** Characteristics of 127 HCC lesions ablated with stereotactic MWA in 88 patients and 90 sessions.

Characteristics	Value
*HCC lesions ablated per patient, n (%)*	
1	61 (67.8)
2	21 (23.3)
3	8 (8.9)
*Mean maximum tumor diameter in mm (range)*	19.9 ± 10.3 (6–53)
*Complications*	
Minor (Grade 1 and 2)	11 (12.2)
Major (Grade 3 to 6)	1 (1.1)
*Tumor size subdivisions, n (%)*	
<30 mm	104 (81.9)
30–50 mm	22 (17.3)
>50 mm	1 (0.8)
*Mean MAM in mm (range)*	4.2 ± 4.2 (0–24)
*Mean follow-up period in months*	25.8 ± 11.6
*Mean time to LTP in months*	12.6 ± 11.4
*Mean LTP rate (%)*	
Per session	41.1
Per ablated HCC	31.0
*OS rates (%)*	
1 st year	98.4
2 nd year	94
3 rd year	90.8

MAM: minimal ablative margin; LTP: local tumor progression; OS: overall survival.

## Data Availability

The source data presented in this study are available on request from the corresponding author. The source data are not publicly available due to patient’s privacy.

## References

[1] European Association for the Study of the Liver (2018). EASL Clinical Practice Guidelines: Management of hepatocellular carcinoma. J. Hepatol..

[2] Voesch S., Bitzer M., Blödt S., Follmann M., Freudenberger P., Langer T., Lorenz P., Jansen P.L., Steubesand N., Galle P. (2022). S3-Leitlinie: Diagnostik und Therapie des hepatozellulären Karzinoms: Langversion 2.0–Juni 2021, AWMF-Registernummer: 032–053OL. Z. Für Gastroenterol..

[3] Beyer L., Luerken L., Radu-Ionita F., Pyrsopoulos N.T., Jinga M., Tintoiu I.C., Sun Z., Bontas E. (2020). Ablation of Hepatocellular Carcinoma. Liver Diseases.

[4] Bale R., Widmann G., Schullian P., Haidu M., Pall G., Klaus A., Weiss H., Biebl M., Margreiter R. (2012). Percutaneous stereotactic radiofrequency ablation of colorectal liver metastases. Eur. Radiol..

[5] Abdullah B.J.J., Yeong C.H., Goh K.L., Yoong B.K., Ho G.F., Yim C.C.W., Kulkarni A. (2015). Robotic-assisted thermal ablation of liver tumours. Eur. Radiol..

[6] Mbalisike E.C., Vogl T.J., Zangos S., Eichler K., Balakrishnan P., Paul J. (2015). Image-guided microwave thermoablation of hepatic tumours using novel robotic guidance: An early experience. Eur. Radiol..

[7] Beyer L.P., Pregler B., Niessen C., Dollinger M., Graf B.M., Müller M., Schlitt H.J., Stroszczynski C., Wiggermann P. (2016). Robot-assisted microwave thermoablation of liver tumors: A single-center experience. Int. J. Comput. Assist. Radiol. Surg..

[8] Harari C.M., Magagna M., Bedoya M., Lee F.T., Lubner M.G., Hinshaw J.L., Ziemlewicz T., Brace C.L. (2016). Microwave Ablation: Comparison of Simultaneous and Sequential Activation of Multiple Antennas in Liver Model Systems. Radiology.

[9] Durand P., Moreau-Gaudry A., Silvent A.-S., Frandon J., Chipon E., Medici M., Bricault I. (2017). Computer assisted electromagnetic navigation improves accuracy in computed tomography guided interventions: A Prospect. randomized clinical trial. PLoS ONE.

[10] Engstrand J., Toporek G., Harbut P., Jonas E., Nilsson H., Freedman J. (2017). Stereotactic CT-Guided Percutaneous Microwave Ablation of Liver Tumors With the Use of High-Frequency Jet Ventilation: An Accuracy Procedural Safety Study. Am. J. Roentgenol..

[11] Beyer L.P., Lürken L., Verloh N., Haimerl M., Michalik K., Schaible J., Stroszczynski C., Wiggermann P. (2018). Stereotactically navigated percutaneous microwave ablation (MWA) compared to conventional MWA: A matched pair analysis. Int. J. Comput. Assist. Radiol. Surg..

[12] Liu Y., Zheng Y., Li S., Li B., Zhang Y., Yuan Y. (2013). Percutaneous microwave ablation of larger hepatocellular carcinoma. Clin. Radiol..

[13] Lin C.C., Cheng Y.T., Lin S.M. (2016). The effectiveness of multiple electrode radiofrequency ablation in patients with hepatocellular carcinoma with lesions more than 3 cm in size and barcelona clinic liver cancer stage A to B2. Liver Cancer.

[14] Xu Y., Shen Q., Wang N., Liu P., Wu P., Peng Z., Qian G. (2017). Percutaneous microwave ablation of 5–6 cm unresectable hepatocellular carcinoma: Local efficacy and long-term outcomes. Int. J. Hyperth. Off. J. Eur. Soc. Hyperthermic Oncol. N. Am. Hyperth. Group.

[15] Bouda D., Barrau V., Raynaud L., Dioguardi Burgio M., Paulatto L., Roche V., Sibert A., Moussa N., Vilgrain V., Ronot M. (2020). Factors Associated with Tumor Progression After Percutaneous Ablation of Hepatocellular Carcinoma: Comparison Between Monopolar Radiofrequency and Microwaves. Results of a Propensity Score Matching Analysis. Cardiovasc. Interv. Radiol..

[16] Ahmed M., Solbiati L., Brace C.L., Breen D.J., Callstrom M.R., Charboneau J.W., Chen M.-H., Choi B.I., De Baère T., Dodd G.D. (2014). Image-guided Tumor Ablation: Standardization of Terminology and Reporting Criteria—A 10-Year Update. Radiology.

[17] Kim Y.-S., Lee W.J., Rhim H., Lim H.K., Choi D., Lee J.Y. (2010). The Minimal Ablative Margin of Radiofrequency Ablation of Hepatocellular Carcinoma (>2 and <5 cm) Needed to Prevent Local Tumor Progression: 3D Quantitative Assessment Using, C.T. Image Fusion. Am. J. Roentgenol..

[18] Nishikawa H., Osaki Y., Iguchi E., Takeda H., Matsuda F., Nakajima J., Sakamoto A., Hatamaru K., Saito S., Nasu A. (2013). Radiofrequency ablation for hepatocellular carcinoma: The relationship between a new grading system for the ablative margin and clinical outcomes. J. Gastroenterol..

[19] Wang X., Sofocleous C.T., Erinjeri J.P., Petre E.N., Gonen M., Do K.G., Brown K.T., Covey A.M., Brody L.A., Alago W. (2013). Margin size is an independent predictor of local tumor progression after ablation of colon cancer liver metastases. Cardiovasc. Interv. Radiol..

[20] Tang H., Tang Y., Hong J., Chen T., Mai C., Jiang P. (2015). A measure to assess the ablative margin using 3D-CT image fusion after radiofrequency ablation of hepatocellular carcinoma. HPB Off. J. Int. Hepato Pancreato Biliary Assoc..

[21] Teng W., Liu K.-W., Lin C.-C., Jeng W.-J., Chen W.-T., Sheen I.-S., Lin C.-Y., Lin S.-M. (2015). Insufficient ablative margin determined by early computed tomography may predict the recurrence of hepatocellular carcinoma after radiofrequency ablation. Liver Cancer.

[22] Hocquelet A., Trillaud H., Frulio N., Papadopoulos P., Balageas P., Salut C., Meyer M., Blanc J.-F., Montaudon M., de Senneville B.D. (2016). Three-Dimensional Measurement of Hepatocellular Carcinoma Ablation Zones and Margins for Predicting Local Tumor Progression. J. Vasc. Interv. Radiol..

[23] Makino Y., Imai Y., Igura T., Kogita S., Sawai Y., Fukuda K., Iwamoto T., Okabe J., Takamura M., Fujita N. (2016). Feasibility of Extracted-Overlay Fusion Imaging for Intraoperative Treatment Evaluation of Radiofrequency Ablation for Hepatocellular Carcinoma. Liver Cancer.

[24] Minami Y., Minami T., Chishina H., Kono M., Arizumi T., Takita M., Yada N., Hagiwara S., Ida H., Ueshima K. (2016). US-US Fusion Imaging in Radiofrequency Ablation for Liver Metastases. Dig. Dis..

[25] Kim S.M., Shin S.S., Lee B.C., Kim J.W., Heo S.H., Lim H.S., Jeong Y.Y. (2017). Imaging evaluation of ablative margin and index tumor immediately after radiofrequency ablation for hepatocellular carcinoma: Comparison between multidetector-row CT and MR imaging. Abdom. Radiol..

[26] Li F.-Y., Li J.-G., Wu S.-S., Ye H.-L., He X.-Q., Zeng Q.-J., Zheng R.-Q., An C., Li K. (2021). An Optimal Ablative Margin of Small Single Hepatocellular Carcinoma Treated with Image-Guided Percutaneous Thermal Ablation and Local Recurrence Prediction Base on the Ablative Margin: A Multicenter Study. J. Hepatocell. Carcinoma.

[27] N’Kontchou G., Mahamoudi A., Aout M., Ganne-Carrié N., Grando V., Coderc E., Vicaut E., Trinchet J.C., Sellier N., Beaugrand M. (2009). Radiofrequency ablation of hepatocellular carcinoma: Long-term results and prognostic factors in 235 Western patients with cirrhosis. Hepatology.

[28] Shiina S., Tateishi R., Arano T., Uchino K., Enooku K., Nakagawa H., Asaoka Y., Sato T., Masuzaki R., Kondo Y. (2012). Radiofrequency ablation for hepatocellular carcinoma: 10-year outcome and prognostic factors. Am. J. Gastroenterol..

[29] Lee D.H., Lee J.M., Lee J.Y., Kim S.H., Yoon J.H., Kim Y.J., Han J.K., Choi B.I. (2014). Radiofrequency ablation of hepatocellular carcinoma as first-line treatment: Long-term results and prognostic factors in 162 patients with cirrhosis. Radiology.

[30] Gardini A.C., Marisi G., Canale M., Foschi F.G., Donati G., Ercolani G., Valgiusti M., Passardi A., Frassineti G.L., Scarpi E. (2018). Radiofrequency ablation of hepatocellular carcinoma: A meta-analysis of overall survival and recurrence-free survival. OncoTargets Ther..

[31] Hermida M., Cassinotto C., Piron L., Aho-Glélé S., Guillot C., Schembri V., Allimant C., Jaber S., Pageaux G.-P., Assenat E. (2020). Multimodal Percutaneous Thermal Ablation of Small Hepatocellular Carcinoma: Predictive Factors of Recurrence and Survival in Western Patients. Cancers.

[32] Facciorusso A., Di Maso M., Muscatiello N. (2016). Microwave ablation versus radiofrequency ablation for the treatment of hepatocellular carcinoma: A systematic review and meta-analysis. Int. J. Hyperth. Off. J. Eur. Soc. Hyperthermic Oncol. N. Am. Hyperth. Group.

[33] Gupta P., Maralakunte M., Kumar M.P., Chandel K., Chaluvashetty S.B., Bhujade H., Kalra N., Sandhu M.S. (2021). Overall survival and local recurrence following RFA, MWA, and cryoablation of very early and early HCC: A systematic review and Bayesian network meta-analysis. Eur. Radiol..

[34] Koo T.K., Li M.Y. (2016). A Guideline of Selecting and Reporting Intraclass Correlation Coefficients for Reliability Research. J. Chiropr. Med..

[35] Laimer G., Schullian P., Jaschke N., Putzer D., Eberle G., Alzaga A., Odisio B., Bale R. (2020). Minimal ablative margin (MAM) assessment with image fusion: An independent predictor for local tumor progression in hepatocellular carcinoma after stereotactic radiofrequency ablation. Eur. Radiol..

[36] Tinguely P., Paolucci I., Ruiter S.J.S., Weber S., de Jong K.P., Candinas D., Freedman J., Engstrand J. (2021). Stereotactic and Robotic Minimally Invasive Thermal Ablation of Malignant Liver Tumors: A Systematic Review and Meta-Analysis. Front. Oncol..

[37] Laimer G., Schullian P., Scharll Y., Putzer D., Eberle G., Oberhuber G., Bale R. (2022). Stereotactic radiofrequency ablation as a valid first-line treatment option for hepatocellular adenomas. Int. J. Hyperth. Off. J. Eur. Soc. Hyperthermic Oncol. N. Am. Hyperth. Group.

[38] Schaible J., Pregler B., Bäumler W., Einspieler I., Jung E.-M., Stroszczynski C., Beyer L.P. (2020). Safety margin assessment after microwave ablation of liver tumors: Inter- and intrareader variability. Radiol. Oncol..

[39] Solbiati M., Muglia R., Goldberg S.N., Ierace T., Rotilio A., Passera K.M., Marre I., Solbiati L. (2019). A novel software platform for volumetric assessment of ablation completeness. Int. J. Hyperth..

